# Single Exhale Biomarker Breathalyzer

**DOI:** 10.3390/s19020270

**Published:** 2019-01-11

**Authors:** Yasha Karimi, Yingkan Lin, Gagan Jodhani, Milutin Stanaćević, Pelagia-Irene Gouma

**Affiliations:** 1 Department of Electrical and Computer Engineering, Stony Brook University, Stony Brook, NY 11794, USA; yasha.karimi@stonybrook.edu (Y.K.); milutin.stanacevic@stonybrook.edu (M.S.); 2 Apple Inc., Cupertino, CA 95014, USA; clarkling@gmail.com; 3 Department of Material Science and Engineering, The Ohio State University, Columbus, OH 43210, USA; gaganjodhani@gmail.com

**Keywords:** metal-oxide gas sensor, breath analysis device, biomarker detection

## Abstract

A single exhale breathalyzer comprises a gas sensor that satisfies the following stringent conditions: high sensitivity to the target gas, high selectivity, stable response over extended period of time and fast response. Breathalyzer implementation includes a front-end circuit matching the sensitivity of the sensor that provides the readout of the sensor signal. We present here the characterization study of the response stability and response time of a selective Nitric Oxide (NO) sensor using designed data acquisition system that also serves as a foundation for the design of wireless handheld prototype. The experimental results with the described sensor and data acquisition system demonstrate stable response to NO concentration of 200 ppb over the period of two weeks. The experiments with different injection and retraction times of the sensor exposure to constant NO concentration show a fast response time of the sensor (on the order of 15 s) and the adequate recovery time (on the order of 3 min) demonstrating suitability for the single exhale breathalyzer.

## 1. Introduction

The medical science has been aware of the diagnostic potential of the human breath for centuries, however even with the recent technological advances, this potential has not been fully realized [1,2]. The breath analysis device is still one of the most promising technologies in the new era of the personalized medicine [3,4,5]. The NO sensor based breathalyzers for home use by patients could have a great impact on management of the airway pathway diseases like asthma. Currently available devices like NIOX Vero [6] are used by medical professionals in their offices thus providing a very limited dataset for asthma management. In addition, these devices are not user-friendly, requiring an extending predefined breathing pattern; this is especially difficult for children. The lack of inexpensive sensor technology that would detect and monitor the concentration of a single gas with high specificity and at low trace concentrations, in the presence of numerous interfering compounds, has presented a main obstacle in advancement of the diagnostics using breath analysis.

The recent work on the design of the breathalyzers has mostly focused on the design of an array of similar sensors and complex signal processing algorithms [7,8,9]. This approach stems from the E-nose technology that had been mostly focused on the classification of odors [10,11,12]. The major drawback of this approach is that most types of gas sensors, including the metal-oxide sensor, suffer from drift in the response and with an array of sensors along with the complex supervised signal processing the calibration of these devices over the longer period of time becomes cumbersome. There have been techniques proposed to combat the drift [13,14,15], however, this still remains the main obstacle in the wider deployment of these systems.

In contrast, we focus on a breathalyzer device with a single metal-oxide sensor specifically sensitive to NO [16,17,18,19]. The sensor used in this work [17] also suffers from a drift in the response over the extended period of time, but due to the cheap manufacturing process, the sensor can be replaced over a shorter period of time after a considerable number of user tests. Replacing the single sensor does not call for supervised recalibration like in the case of an array of sensors. The sensitivity of the γ-phase of WO${}_{3}$ metal-oxide sensor to NO, selectivity and response in the presence of different levels of humidity has been studied extensively [16,20,21,22]. However, the stability of the sensor response over time and response time has not been studied previously.

For integration of the sensor into a breathalyzer, a circuit for the conversion of the resistance of the sensor, that is proportional to the NO concentration to a digital value, is required. There is a wide range of proposed instrumentation techniques developed for resistance measurement in the literature [23,24], with the readout ICs that achieve the accuracy down to 0.1% by tracking baseline resistance [25,26]. In the designed prototype, we opted for the discrete implementation of the front-end readout circuit that achieves satisfactory precision for wide range of measured resistances. The power consumption could be significantly reduced using integrated implementation and we envision that our next generation prototype would have application specific integrated circuit (ASIC) for resistance measurement, along with the sensing and control of the temperature of the sensor [27].

The paper is organized as follows. In Section 2 we describe the preparation of the sensor and experimental setup for gas delivery and data acquisition. Section 3 provides a detailed description of the front-end readout circuit that interfaces the sensor along with the results of the experimental characterization of the sensor. The concluding comments are in Section 4.

## 2. Experimental Method

A NO sensor was prepared according to the procedure described in [17]. The materials were synthesized by flame spray pyrolysis method using lab-scale nanopowders production system from Tethis (NP10) in our lab. The NP10 system contains three main parts: nozzle unit, dispensing system and control unit, which are all located in the lab vented fume hood. The system is controlled by a computer, which guarantees the safety and accuracy of the synthesis process. A collecting system is based on glass fiber filters on the top of the collecting chamber.

The precursor solution using is prepared in the following way: 0.3 M of tungsten (VI) isopropoxide (99% All-Chemie) was dissolved in 2-propanol in nitrogen atmosphere glove box. After aging for a day, the precursor was supplied at a rate of 5 mL/min through the flame nozzle and dispersed by oxygen with a rate of 5 slm (standard liter per minute) to form fine spray. The fine spray was ignited and supported by the flame that was combustion of methane and oxygen at the rate of 1.5 slm and 3.0 slm respectively. The synthesized particles were deposited beneath the glass fiber filter (Whatman) and collected after the process was done.

**Figure 1:**
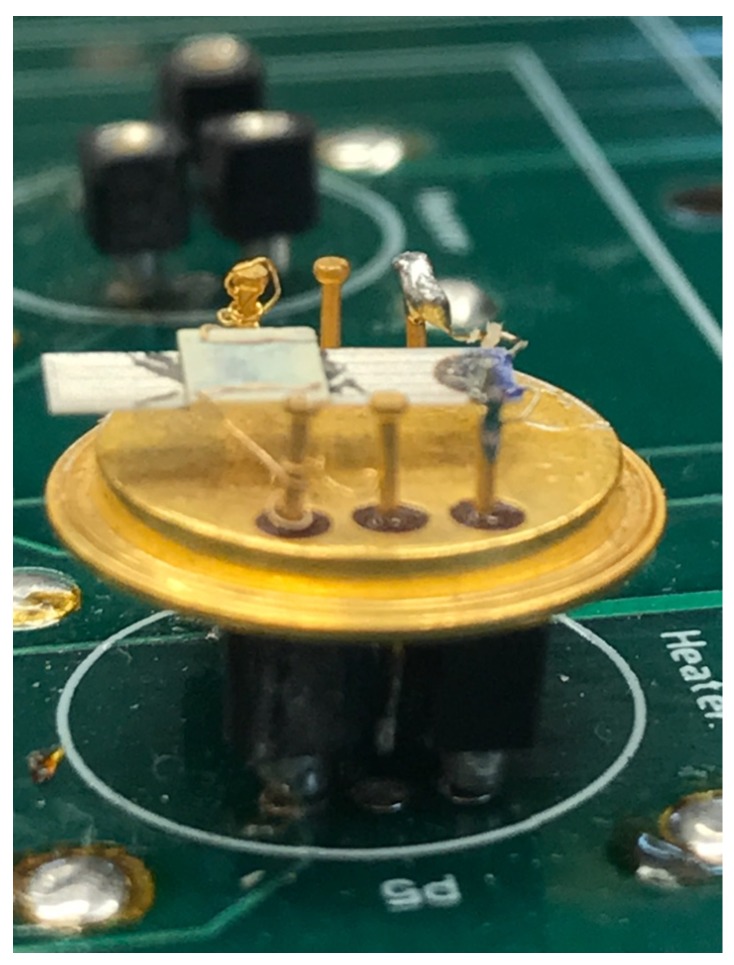
Sensor and heater assembly.

The sensor was connected to TO-8 package through gold wires (Alfa Aesar, 0.25 mm diameter, 99.998%) bonded on the integrated platinum circuits on the Al${}_{2}$O${}_{3}$ substrates, as shown in Figure 1. The heater was mounted below the sensor in the same package and adhered to the sensor using alumina paste to improve the heat conduction. The operating temperature of the sensor throughout the experiments was kept at 200 °C.

For assessing the stability of and for the calibration of the NO sensor response, we designed the data acquisition system that can perform simultaneous measurement of the response of three different gas sensors. The system comprises the printed circuit board (PCB) with a gas flow chamber that houses three sensors along with the circuits for readout of the sensor signal and for control of the temperature of the heater. The chamber with three sensors has been used to enable experiments that could quantify the difference in performance of the breathalyzer containing either a single sensor or an array of sensors. An array of selective sensors can be used to account for interference from two different gases that can slightly influence the NO sensor response.

The chamber in the data acquisition system was designed to mimic the chamber that could be used in a single exhale breathalyzer. The dimensions of the teflon chamber are 3.5 cm × 9.5 cm × 2.5 cm. The chamber has an inlet for the incoming gas and an outlet for the outgoing gas. The inlet is designed to also enable injection of the gas into the chamber through Tedlar gas sampling bags. The front-end readout electronics, that performs the conversion of the sensor resistance to voltage signal and the circuit for the control of the temperature of the heater is designed on the board. This circuitry is directly translated into the design of the wireless handheld prototype. The output voltages of the front-end readout electronics are interfaced to National Instruments data acquisition card (NI-DAQ), NI 6259, and converted to digital domain. The temperature of the heater is also controlled through NI-DAQ.

**Figure 2:**
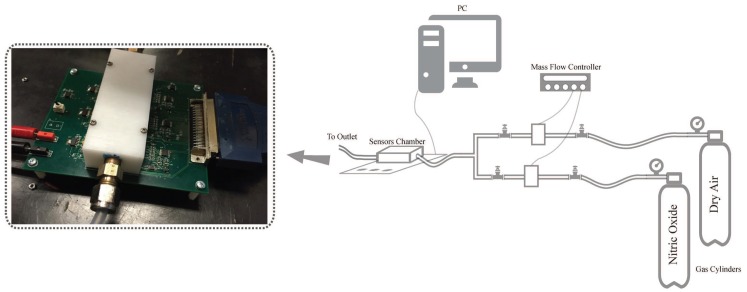
Experimental setup for gas delivery along with the data acquisition board.

To generate the precise concentration of nitric oxide, the setup shown in Figure 2 was used. The gases we used were UHP Nitrogen, UHP Oxygen, and 10 ppm nitric oxide in nitrogen (all of them were from Global Calibration Gases). Concentration of nitric oxide was controlled by varying the flow rates in conjunction with nitrogen and oxygen flow rates. The flow rates of the gases were controlled by a type 247-MKS 4-channel readout and 1479 MKS mass flow controllers in the unit of sccm (standard cubic centimeter per minute). The flow rate in all experiments was set at 250 sccm.

The gas lines were connected to the inlet of the gas chamber on three-sensor PCB. The following pattern of the gas injection was used in the experiments. The gas was injected for injection time $t_{inj}$ at the specific concentration and the time between the consecutive injections was retraction time $t_{ret}$. The concentration of NO over the sensor was constant over the injection time. The resistance of the sensor was measured using the on-board front-end circuit for conversion of the sensor resistance to voltage signal. The output voltage of the front-end readout circuit was recorded with 16-bit ADC integrated on PCI data acquisition card at 100 Hz sampling rate and stored on PC.

### Calibration Procedure

The systematic sources of error in the measurement of the sensor resistance are the imprecision in the value of the feedback resistor, the voltage offset of the operational amplifier and the current offset through a feedback resistor stemming from the leakage currents of the switches and the input current of the operational amplifier. In order to minimize the effect of finite resolution of the feedback resistor, very high precision resistors could be used. However, in the higher ranges of the feedback resistors, for the values of the feedback resistor of 450 kΩ, 4 MΩ, and 33 MΩ, very low tolerance resistors are prohibitively expensive. In the lower range, 4.99 kΩ and 49.9 kΩ, low price resistors with 0.01% tolerance are common.

We perform calibration procedure before a new gas sensor is placed in the prototype in order to estimate the values of the feedback resistors and voltage and current offsets. For the calibration, we employ two low-tolerance resistors and a high-resolution digital-to-analog (DAC) converter. The first low-tolerance resistor R${}_{ref1}$ matches the feedback resistor in the lowest resistance measurement range. In the first calibration measurement, the output voltage is measured without the sensor with only the switches in the branches with R${}_{f1}$ and R${}_{ref1}$ being turned on. The deviation from the ideal output voltage in this range would be dominated by the voltage offset of the operational amplifier, while the effect of the current offset and the finite resolution of the feedback resistor can be neglected.

## 3. Experimental Results

### 3.1. Readout Front-End

The gas sensor (see Figure 1) can be modeled as an electrical resistance. The concentration of the selected gas over the fabricated sensor is quantified by measurement of the sensor resistance. The electrical model can also incorporate the parasitic capacitance of the sensor, which in the proposed readout circuit does not affect the quantification of the resistance value. The total sensor resistance $R_{s}$ is superposition of the two resistances, a baseline resistance $R_{b}$ and sensor resistance proportional to the concentration of the selected gas $R_{g}$. The baseline sensor resistance is the resistance of the sensor when the selected gas is not present in the environment. The baseline resistance drifts over time and affects the sensitivity of the measurements. A calibration procedure has to be incorporated in the system to compensate for this variation.

**Figure 3:**
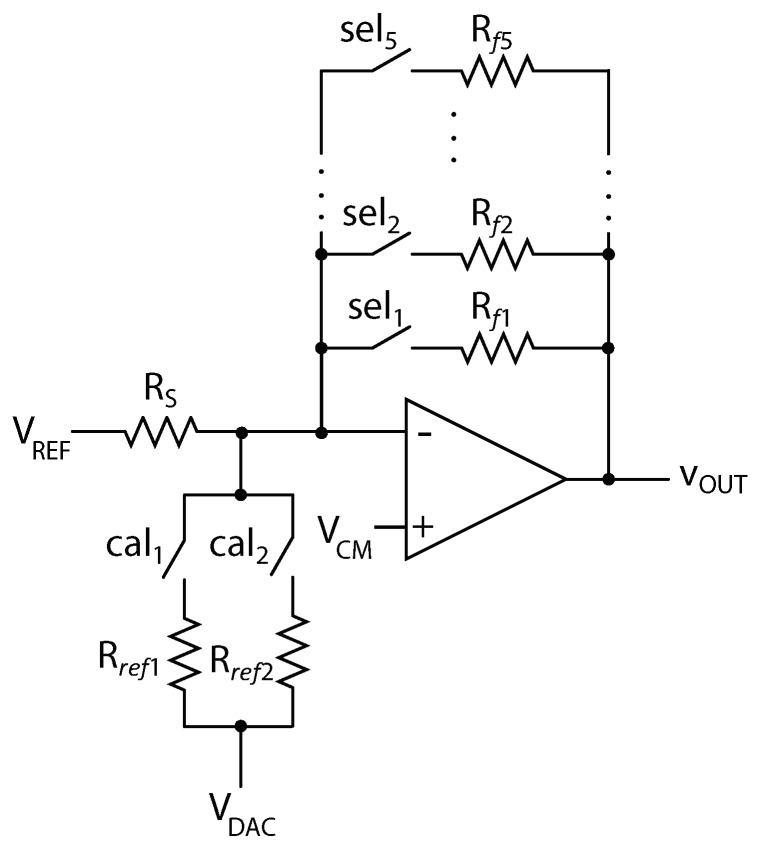
Front end readout circuit.

The front-end readout circuit comprises the programmable transresistance amplifier [23,25], that converts the resistance of the gas sensor to voltage, and is followed by the second-order low-pass filter. The transresistance amplifier is shown in Figure 3 and the operational amplifier used is AD8505. For a specific sensor operating at a specific temperature, we expect that the variation of the sensor resistance is at most two orders of magnitude. However, as the designed readout circuit is intended to be used with different sensors that can operate at different temperatures, we assume that the resistance of the sensor $R_{s}$ can change over a wide range, from 2 kΩ to 100 MΩ. This corresponds to the range of 5 decades. In the transresistance amplifier, the output voltage is non-linear function of the sensor resistance:

(1) $$V_{out}=V_{ref}+\frac{R_{f}}{R_{s}}\left(V_{cm}-V_{ref}\right)$$

where $R_{f}$ is the feedback resistance, $V_{cm}$ is the reference voltage at the non-inverting input of the operational amplifier and $V_{ref}$ is the reference voltage at one of the outputs of the sensor. The voltages $V_{cm}$ and $V_{ref}$ are set to 1.5 V and 0.5 V. The sensitivity of the conversion depends on the resistance value:

(2) $$\frac{dV_{out}}{dR_{s}}=-\frac{R_{f}}{R_{s}^{2}}\left(V_{cm}-V_{ref}\right)$$

As the output voltage has limited range to cover the whole range of the sensor resistance, the feedback resistor $R_{f}$ is programmable and the gas resistance is measured across different ranges set by the value of the feedback resistor. The number of different feedback resistors and their values is set in order to achieve the resolution of 12-bits in the measurement of the gas resistance over the full range. In a single measurement range, the minimum value of the sensor resistance with respect to the value of the feedback resistance that can be observed is defined by the maximum output voltage of the operational amplifier

(3) $$\frac{R_{s}^{min}}{R_{f}}=\frac{V_{cm}-V_{ref}}{V_{out}^{max}-V_{cm}}$$

Since the used op-amp has a rail-to-rail output voltage, we assume that the maximum output voltage $V_{out}^{max}$ is 4.5 V. The maximum value in a single measurement range is set by the desired resolution of the resistance conversion, as the sensitivity is minimum for the maximum value of the sensor resistance. The maximum resistance that can be measured in specific range $R_{s}^{max}$ with respect to the feedback resistor is expressed as the function of the resolution of the conversion of the sensor resistance $S_{Rs}$

(4) $$\frac{R_{s}^{max}}{R_{f}}=\frac{V_{cm}-V_{ref}}{V_{lsb}}S_{Rs}$$

where $V_{lsb}$ is the voltage of the least-significant bit in 16-bit analog-to-digital conversion (ADC) of the output voltage assuming that ADC has a full range of 5 V. For the chosen values of the reference voltages, the ratio of the minimum sensor resistance to feedback resistor is 1/3, while the ratio of the maximum sensor resistance to feedback resistor for 12-bit resolution is 3.2.

**Figure 4:**
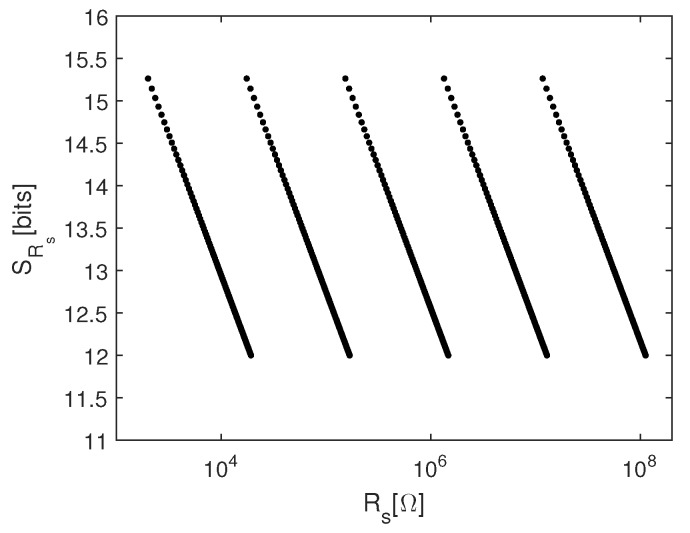
Resolution of the sensor resistance measurement.

When the value of the gas resistor reaches the limit of the particular range, the value of the feedback resistor is changed. To guarantee the continuity of the measurement, the ranges are designed to have 10% overlap. To cover the full range of the sensor resistance and to maintain 12-bit resolution, there are five different values that the resistor $R_{f}$ can take. The chosen values of the feedback resistor are 4.99 kΩ, 49.9 kΩ, 450 kΩ, 4 MΩ, and 33 MΩ. The resolution of the sensor resistance measurement over the full range is shown in Figure 4.

The low-pass filter following the transresistance amplifier, that converts the sensor resistance into a voltage signal, is implemented as the second-order unity-gain Sallen-Key filter. The cut-off of the filter is 19 Hz. 

#### 3.1.1. Calibration

From the measured output voltage, a value of the voltage offset is estimated. The second low-tolerance resistor R${}_{ref2}$ has the value of 100 kΩ with 0.01% tolerance. We perform two measurements of the output voltage in the highest sensor resistance measurement range with only the switches in the branches with R${}_{f5}$ and R${}_{ref2}$ being turned on. From these two values, we obtain estimates of the current offset and the deviation of the resistor R${}_{f5}$ from the ideal value. The single output voltage measurements in two other resistance ranges, provide estimates of the deviation of the resistors R${}_{f3}$ and R${}_{f4}$ from their ideal values. The performance of the readout circuit within each scale was verified using high-precision resistors. During measurements with the proposed sensor, the readout circuit successfully changed the scales when an end of the scale was reached, demonstrating the stability of the developed circuit.

#### 3.1.2. Noise

The dominant noise sources in the front-end readout circuit are the thermal noise from the gas sensor resistance and the feedback resistance, as well as the noise from the operational amplifier. The output noise at the transresistance amplifier that stems from the resistances is:

(5) $${\bar{v}}_{nR}^{2}=\int 4kTR_{f}\frac{R_{f}+R_{g}}{R_{g}}df$$

while the noise contribution from the operational amplifier is:

(6) $${\bar{v}}_{nop}^{2}=\int v_{n}^{2}{\left(\frac{R_{f}+R_{g}}{R_{g}}\right)}^{2}df,$$

where $v_{n}$ denotes the input noise of the operational amplifier. The rms value of the total noise obtained at the output of the low-pass filters is the highest for the largest feedback resistor and is equal to 18 μV.

### 3.2. Characterization Experiments Using Data Acquisition System

Sensor characterization experiments were performed using the fabricated sensor and the described data acquisition system along with the setup for gas delivery. As the selectivity of the sensor toward NO has been previously demonstrated [17], in the described experiments we characterize the reliability of the sensor response, along with the response and recovery times. These experiments were performed in order to quantify the long-term variability and stability of the sensor response. The main factor that affects the stability of the response is the drift in the baseline resistance.

In all of the performed experiments the resistance of the sensor was reaching baseline value for the specific temperature in less than a minute. We observe the baseline of the resistor as the average value of the sensor resistance for 0.1 s before the injection of the gas. The observed value of the sensor resistance as the resistance change proportional to the gas concentration is observed as the difference between the baseline resistance and the peak value of the sensor resistance in a single injection time window. The sensor response is then normalized as the ratio of the observed resistance change and the baseline resistance.

#### 3.2.1. Reliability of the Sensor Response

**Figure 5:**
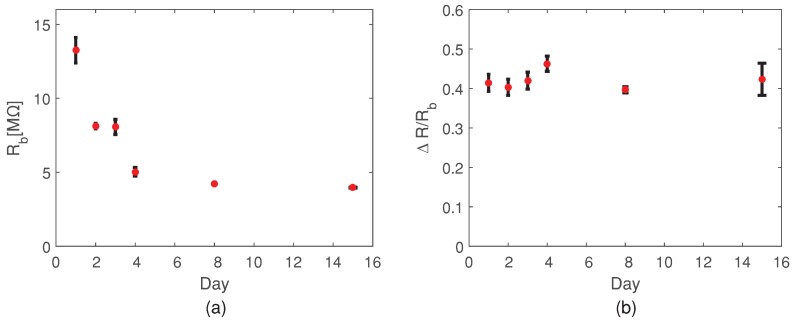
(**a**) Baseline resistance of sensor over time period of two weeks; (**b**) sensor response over time period of two weeks for NO concentration of 200 ppb.

To determine the reliability and robustness of the sensor response, we perform measurements of the gas concentration of 200 ppb over a period of two weeks. At each specific day, 6 measurements of 200 ppb concentration were performed, two at a time. Before each measurement the sensor was heated to 200 °C and after the measurement the temperature of the sensor returned to the room temperature. In Figure 5a, we present the value of the baseline resistance over the two weeks. We can notice that the value of the baseline resistance is reducing over the first few days, while it gets stable after a week, with low variability in the daily measurements.

In the Figure 5b, we show the variability of the sensor response for the constant NO concentration of 200 ppb over the period of two weeks. We can observe the independence of the normalized sensor response with respect to the variation in the baseline of the sensor.

#### 3.2.2. Characterization of Sensor Response Dynamics

We perform characterization study of the dynamics of the sensor response to determine the feasibility of the sensor use in a single exhale breathalyzer. The sensor has to respond quickly to the change in NO concentration in a single exhale breath, as the exhaled gas is briefly captured in the gas chamber. We should also be able to use the breathalyzer for consecutive measurements and in that case the recovery time of the sensor should be short. The initial estimates of the response and recovery times are obtained from experiments with extended injection and retraction times and in the experiments described we use these estimated times to find the limits of both response and recovery times.

**Figure 6:**
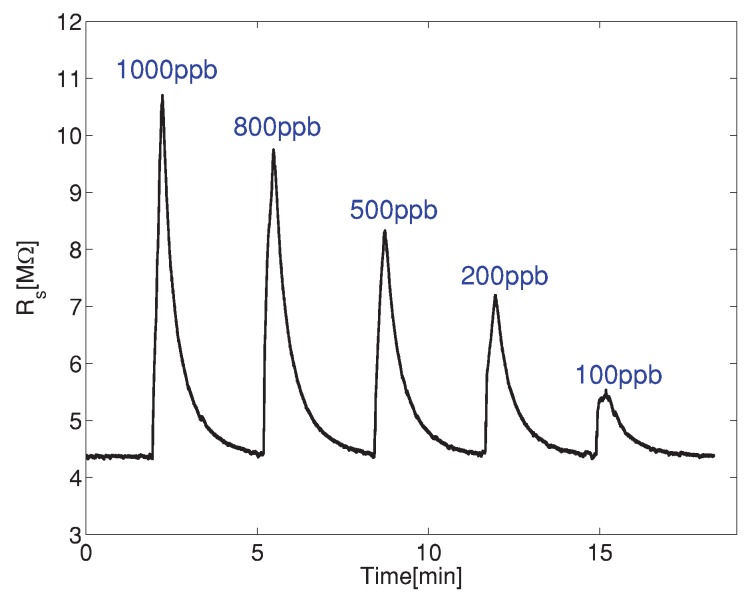
The time response of the gas sensor with t${}_{inj}$ = 30 s and t${}_{ret}$ = 3 min for 5 different concentrations of nitric oxide.

To determine the response time of the sensor, we performed two experiments with different injection time of the gas, t${}_{inj}$ = 15 s and t${}_{inj}$ = 30 s. In both cases, the retraction time t${}_{ret}$ is equal to 3 min. In Figure 6, we show the time response of the sensor, with the NO concentrations of 100 ppb, 200 ppb, 500 ppb, 800 ppb and 1000 ppb for the second case, t${}_{inj}$ = 30 s and t${}_{ret}$ = 3 min.

**Figure 7:**
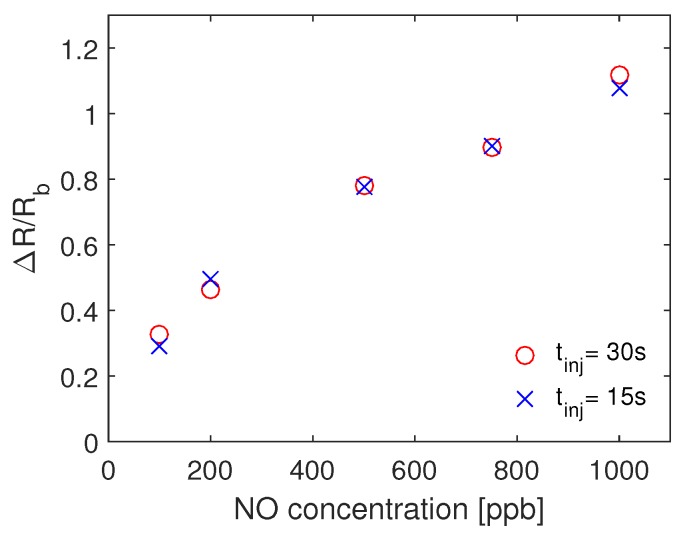
The mean of the response of the sensor for different concentration of NO when t${}_{inj}$ = 15 s and t${}_{inj}$ = 30 s. The retraction time in both cases is t${}_{ret}$ = 3 min.

To compare the effect of the injection time, and characterize the response time of the sensor, we plot in Figure 7 the mean value of the sensor resistance for each of the used concentration for two different injection times. The experiments for each injection time were repeated three times on three different days. As the difference between the two responses at all the concentrations is small, we can conclude that the response time of the sensor is lower than 15 s.

**Figure 8:**
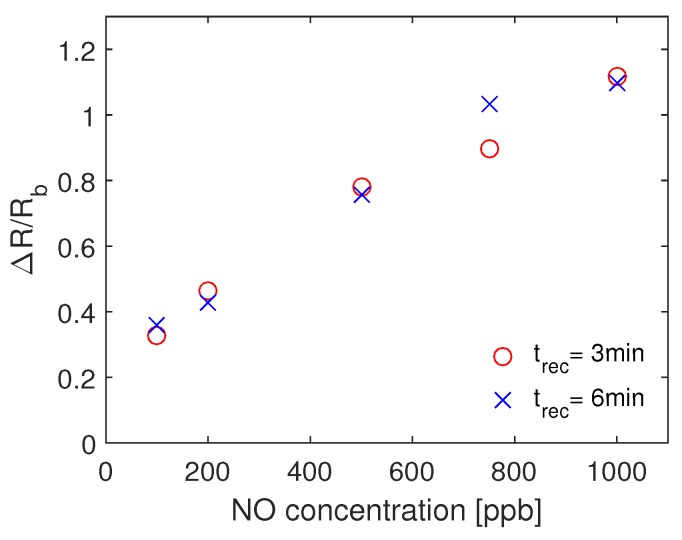
The mean of the response of the sensor for different concentration of NO when t${}_{ret}$ = 3 min and t${}_{ret}$ = 6 min. The injection time in both cases is t${}_{inj}$ = 30 s.

The recovery time, the time which is required for the sensor response to return to the baseline resistance, sets the time interval between consecutive samples so the single exhale prototype can be performed. We compared the difference between the sensor recovery time by conducting experiments with different retraction time, t${}_{ret}$ = 3 min and t${}_{ret}$ = 6 min, Figure 8. As the difference between response of the sensors in both cases is small, we can conclude that the recovery time of the sensor is lower than 3 min.

#### 3.2.3. Discussion

We have demonstrated the stability of the sensor response over the period of two weeks. The minimum injection and retraction times were experimentally obtained and demonstrate the suitability of the sensor for the single exhale breathalyzer. The two amperometric sensors with the similar response time and sensitivity to NO have been presented in the literature [28,29], however, long-term reliability studies of these sensors have not been published.

## 4. Conclusions

Allowing individuals to identify and monitor specific biomarkers of disease in their own breath is one of the anticipated benefits of personalized medicine. This knowledge may be used for the early detection of disease, better control of metabolic malfunctions, or to assess the efficacy of or fine tune the delivery of a therapeutic treatment as needed. Such effortless non-invasive testing, like capturing and analyzing a single exhaled breath, is expected to revolutionize public health screening. The breath analyzer technology described here is based on a nanosensor device that detects nitric oxide in breath. NO is a biomarker that the medical literature has associated with certain medical conditions, such as asthma. It takes seconds to give out the reading of the gas concentration. The principle of their operation is resistive chemosensing; once the gas of interest selectively interacts with the ‘sensing’ material, it changes its electrical resistance. Universal use of the nanosensor breathalyzer is anticipated based on the fact that everyone, from prematurely born infants to the aging and incapacitated population, from the servicemen in a battlefield to the underprivileged sick, may easily provide an exhaled breath sample. This nanosensor technology may analyze the breath exhale in a reliable, affordable (really cheap), fast, and easy way on-site, at the point where care is actually needed. At home, at work, on the road, in the field, at a hospital, or at a doctor’s office, that is to say, anywhere and everywhere.
